# Gene Therapy for Retinal Degenerative Diseases: Progress, Challenges, and Future Directions

**DOI:** 10.1167/iovs.64.7.39

**Published:** 2023-06-30

**Authors:** Selina Drag, Farokh Dotiwala, Arun K. Upadhyay

**Affiliations:** 1 Ocugen, Inc., Malvern, Pennsylvania, United States

**Keywords:** gene therapy, retina, viral vector, immunogenicity, gene therapy challenges

## Abstract

Since long before the first approval of gene therapy for retinal disease, ocular gene therapy has captured the hopes of patients, clinicians, and scientists alike. Indeed, the retina provides a unique system for studying and treating ocular diseases, and it holds the distinction as the first tissue targeted by an approved gene therapy for inherited disorders in the United States. There are many methods for addressing genetic diseases in the eyes using a wide range of potential delivery systems and vectors. However, despite the immense progress over the last several decades, both old and new challenges remain, such as the long-term effects of treatments, immunogenicity, targeting, and manufacturing. This review provides a discussion of the history of ocular gene therapy, the various gene therapy approaches, methods to deliver a gene directly to ocular tissues (including both routes of administration and vectors), challenges to ocular gene therapy, the current clinical trial landscape, and future directions of the field.

Genetic diseases were once formidable and devastating because, until recently, they were considered untreatable.^1^ Gene therapy has provided a unique opportunity to treat and even cure these diseases, offering hope to the millions of people either affected by inherited disorders or carrying disease-causing mutations.^2^ Its history is rich and complicated, dating back 7 decades to the first observations of viral gene transfer, and has involved great scientific advancements, including improvements in vectors and vector design, and complications, such as serious adverse events and patient deaths.^3^^,^^4^ The earliest gene therapies used several different viral vector-based platforms, and both adenovirus and lentivirus were shown to effectively transduce retinal cells.^5^^–^^8^ However, 2 clinical studies in the late 1990s suffered serious adverse events: cytokine storms, multiple organ failures, T-cell leukemia, oncogene activation, and even death.^3^^,^^4^ These complications slowed the progress of research and development as researchers looked for answers and solutions to these risks. New technologies continue to provide innovative new treatment platforms, such as the clustered regulatory interspaced palindromic repeats (CRISPR)/CRISPR-associated protein 9 (Cas9) system and optogenetics.^9^^,^^10^

Luxturna from Spark Therapeutics, for the treatment of Leber congenital amaurosis (LCA) type 2, showed safety and efficacy in phase I/II clinical trials^11^^–^^13^ and was the first US Food and Drug Administration (FDA)-approved gene therapy (2017) for an inherited disorder in the United States.^14^^,^^15^ Yet, despite this landmark achievement, no other ocular gene therapies have been approved in the United States. This may be in part due to the biological challenges to efficient transduction and minimizing immunogenicity or the practical challenges in scaling up manufacturing and reaching the target tissues.^16^^–^^18^ Improvements to production methods, administration, and drug design and targeting can all influence the success of gene delivery to the retina in future trials, and ocular gene therapy studies thus far have demonstrated the potential for success.^18^

The retina has long been considered an excellent target for gene therapy. It offers advantages, such as an enclosed, immune-privileged site protected by the blood-retina barrier.^19^ Due to the small size of the retina and a lack of cellular proliferation in adulthood, only small amounts of the vector are required to treat retinal diseases.^15^ Already established surgical procedures or clinical practices can be used to administer ocular gene therapy products.^1^^,^^15^ Easy real-time ocular monitoring by optical coherence tomography and fundus imaging allows monitoring the drug effects in both animal models and clinical trial participants.^15^

Despite these advantages, emerging evidence suggests that sites, such as the eye and central nervous system, once considered immune privileged, are more vulnerable to adverse immune reactions than previously believed.^20^^–^^22^ Several studies have observed antidrug antibody responses and inflammation in the eyes due to immune cell infiltration following retinal gene therapy.^21^^,^^23^^–^^27^ Continuing work in improving vector systems may offer alternatives to the current popular systems for gene delivery.^9^^,^^27^^–^^29^

## History of Gene Therapy Development for Retinal Diseases

Although the history of gene therapy goes back to the 1950s, retinal gene therapies have been studied only since the 1990s. In 1994, the first attempts to identify a vector for retinal gene delivery occurred, using adenovirus-based systems in mice.^5^^,^^6^ These studies showed efficient transduction of the retinal pigmented epithelium (RPE) via subretinal injection; however, photoreceptor transduction was refractory and required higher treatment doses. In 1996, Bennett et al. went on to show successful gene therapy using an adenoviral vector in the *rd1* mouse model of recessive retinal degeneration.^30^ Around that same time, other groups used an HIV-based lentiviral or adeno-associated virus (AAV)-based vectors to demonstrate efficient photoreceptor and RPE transduction.^7^^,^^8^ These studies also noted that younger animals treated before the onset of degeneration demonstrated improved gene transfer to photoreceptors.^15^

**Figure 1.**
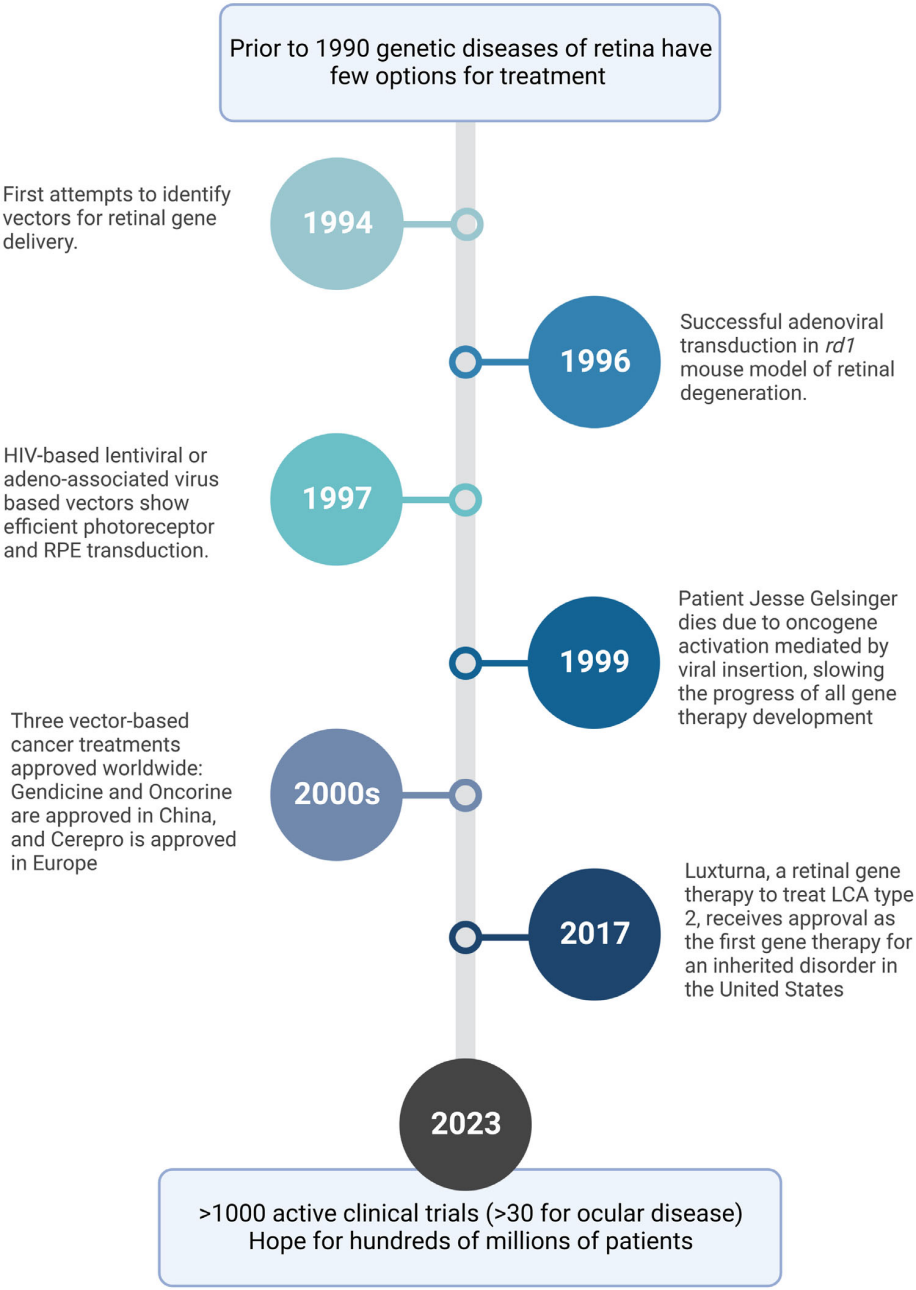
History of the development of retinal gene therapy (created with BioRender.com).

In 1999, gene therapy in general suffered a setback due to the tragic death of the gene therapy patient, Jesse Gelsinger, who was enrolled in a gene therapy trial to treat ornithine transcarbamylase deficiency. Later, 5 of 20 patients with severe combined immunodeficiency (SCID)-X1, treated with retrovirus gene therapy, developed T-cell leukemia within 2 to 6 years following treatment.^3^^,^^4^ These tragedies slowed gene therapy development as researchers, clinicians, and regulators grappled with the risks associated with this technology. Despite the serious setbacks in these clinical trials, advancements continued, with the first gene therapy products Gendicine, Oncorine, and Cerepro being approved for clinical use.^4^^,^^31^ An overview of the history of retinal gene therapy is provided in Figure 1.

## Gene Therapy Approaches for Retinal Diseases

**Figure 2.**
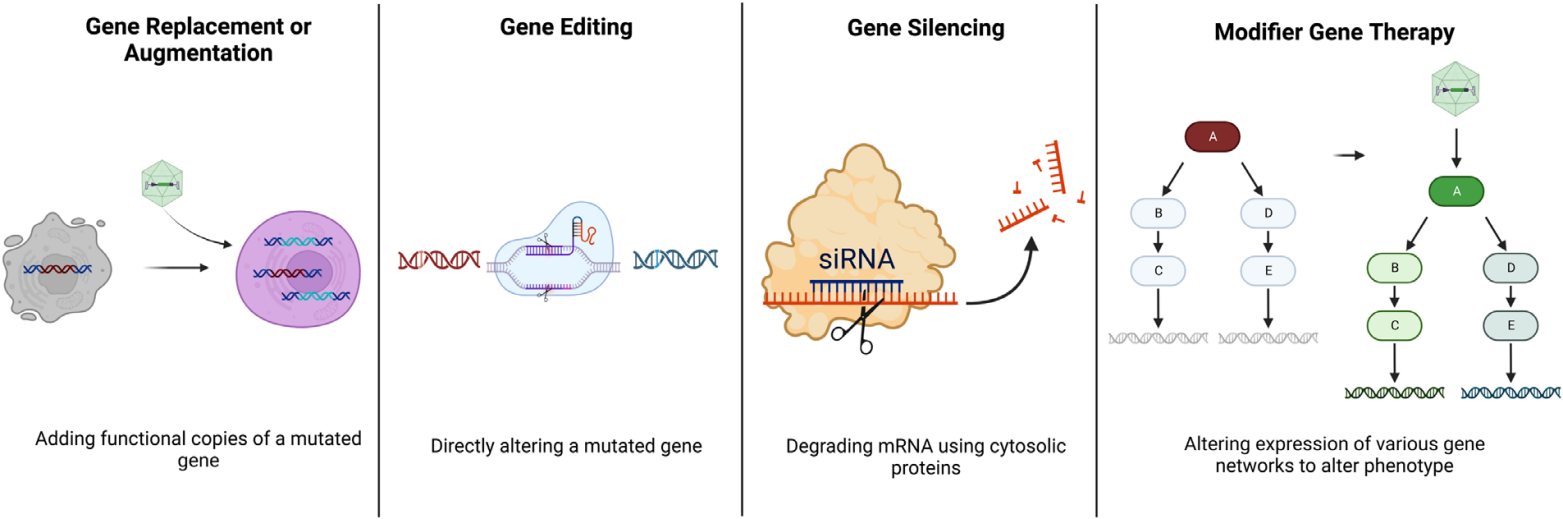
Gene therapy approaches. There are four major approaches to gene therapy (left to right). Gene replacement or augmentation is when a functional copy of a damaged, non-functional gene is added to augment the production of functional protein. In gene editing, mutations in a gene are corrected or expression of the mutated protein is reduced to alter a diseased state. Gene silencing uses the RNAi mechanism to eliminate the aberrant expression of the targeted pathogenic protein in acquired diseases. Modifier gene therapies provide a modifier gene that can affect pathways downstream or upstream from the damaged gene, allowing for the expression of multiple genes to be altered with a single treatment (created with BioRender.com).

Retinal gene therapy approaches vary based on the nature of the mutation (Fig. 2, Table 1) and maybe gene replacement/augmentation, silencing/editing the mutated gene, or supplying a gene that affects the upstream or downstream pathways from the defective gene to improve cellular function (as in modifier therapy). Retinal gene therapies include the use of diverse vectors (Table 2) and routes of administration (Table 3).

**Table 1.** Gene Therapy Approaches

Approach	Description	Advantages	Limitations	When can it be Used?	Examples
Gene Replacement	Addition of a functional copy to augment or replace a dysfunctional gene (single gene and single therapy)	Straightforward, simple approach	The genetic mutation must be known. Not useful for polygenic diseases Expensive to produce a single gene therapy for every affected gene	Monogenic recessive inherited disorders Genetic basis of a disease is known	Luxturna (voretigene neparvovec-rzyl, Spark Therapeutics, Inc.)
Gene silencing	Use of siRNA to degrade sequence-targeted mRNA, effectively removing the product of a defective gene	Employs a natural process to reduce aberrant gene expression	RNA is unstable and subject to degradation in cells. siRNA has poor bioavailability. Non-specific targeting may cause off-target effects and immunogenicity	Inherited diseases that involve overexpression of pathogenic genes or inherited dominant mutant forms.	4D-150 (4D Molecular Therapeutics) for wet AMD RGX-314 (REGENXBIO Inc.) uses AAV8 to encode for anti-VEGF Fab QPI-1007 siRNA (Quark Pharmaceuticals) for non-arteritic anterior ischemic optic neuropathy Tivanisiran (Sylentis) for ocular pain and dry eye disease
Gene editing	Correction of individual mutations or reducing expression of a mutated protein in a targeted manner	Directed, specific approach to address mutations. Permanent, precise modification to DNA	The genetic mutation must be known. Not useful for polygenic diseases Expensive to produce a single gene therapy for every affected gene	Monogenic disorders- point or small mutations, splicing defects Genetic basis of the disease is known	EDIT-101 (CRISPR/Cas9, Editas Medicine)
Modifier gene therapy	Modifier genes are used to affect pathways upstream and downstream of a damaged gene	Mutation-agnostic approach Multiple genes are affected with a single treatment	Identifying appropriate modifier genes for a specific disease phenotype	Polygenic and multifactorial genetic diseases	OCU400 (AAV5-h*NR2E3*, Ocugen, Inc.)

### Gene Replacement Therapy

The gene replacement is a direct approach that supplies a functional copy of a damaged or nonfunctional gene to augment the production of functional protein. This therapy addresses the missing role of the damaged gene without changing it. Gene replacement is best suited for monogenic recessive inherited diseases, for example, mutations in the *CEP290* gene, is one of the most common causes (15–20%) of LCA.^32^ Luxturna (Spark Therapeutics, Inc.) provides a functional *RPE65* gene to patients with LCA with *RPE65* mutations (5–10%). However, despite the apparent success of Luxturna in the clinic, there has been some question regarding its durability of effect and effectiveness across patients with different genetic backgrounds, as well as the limitations, such as a need for remaining tissue prior to treatment and deciding how to quantify the effects of treatment.^33^ Several other products using gene replacement therapy are also in clinical trials (Table 4).

Retinal diseases are caused by a wide range of mutations in many different genes with varied inheritance patterns, such as autosomal dominant, autosomal recessive, and X-linked. For example, in retinitis pigmentosa (RP), more than 3000 mutations in approximately 70 genes have been implicated in disease pathogenesis, and, in many cases of RP, a genetic basis cannot be identified.^34^ In addition, not all gene mutations can be addressed using a gene replacement approach, including dominant mutations, large genes which cannot be packaged in currently utilized delivery vectors, and polygenic conditions. Gene replacement for each mutation would prove costly and limit patient access.

### Gene Silencing

Gene silencing with small interfering RNA (siRNA) or microRNA (miRNA) that target VEGF, are currently in development for the treatment of age-related macular degeneration (AMD), glaucoma, and several other ocular diseases.^35^^–^^38^ Several clinical trials using targeted gene silencing techniques are in progress (Table 4).^39^^–^^41^ However, no clinical trials for this approach in the ocular space have progressed past phase III, as this technique faces several challenges, such as RNA instability, poor bioavailability, and nonspecific targeting leading to off-target effects.^35^

### Gene Editing

In gene editing, mutations in a gene are corrected or the expression of the mutated protein is reduced to alter a diseased state. Several gene editing techniques have been developed, including CRISPR/ Cas9, transcription activator-like effector nucleases (TALENs), zinc finger nucleases (ZFNs), and homing endonucleases or meganucleases.^42^ Of these, the most well-known gene editing technique is the CRISPR/Cas9 system has shown potential in gene therapy. CRISPR/Cas9 is a two-component system that involves a guide RNA specific to the gene of interest and an endonuclease that induces a site specific double-stranded DNA to allow for genetic modification.^9^ This allows for a permanent and precise modification or removal of the mutation associated with a particular disease.^43^ While addressing mutations in a single gene, CRISPR is not effective to patients without a known genetic diagnosis.

Gene therapies using CRISPR/Cas9 technology are in clinical trials (Table 4). In the retinal space, EDIT-101 (Editas Medicine, NCT03872479) is a gene therapy to treat LCA type 10, targeting the IVS26 mutation that causes improper splicing between exons 26 and 27 leading to a prematurely truncated, nonfunctional *CEP290* gene. This treatment uses CRISPR/Cas9 to cause deletions or inversions of the IVS26 mutation both of which restore correct splicing and CEP290 function.^43^^–^^45^ Prime editing (PE) is an upcoming technique that uses reverse transcriptase and Cas9 to repair genome mutations with the potential to correct multiple mutations as well as small insertions and deletions.^43^ However, although this is a promising technique for the future treatment of genetic disorders, it still faces some of the same challenges as traditional gene therapy, particularly the sheer volume of genes involved in disease pathogenesis.

### Modifier Gene Therapy

The existence of modifier genes, or genes that may affect the expression of other genes, particularly mutant genes, without affecting healthy phenotypes, has been known since the early 1940s,^46^ and development of therapies to apply these modifier functions to disease has grown rapidly. The severity of the phenotype caused by some gene mutations can be influenced by these modifier genes.^47^^–^^49^ Modifier genes can affect pathways downstream or upstream from multiple defective genes, thereby addressing a clinical phenotype without the need for a genetic diagnosis and in a mutation-agnostic manner. Modifier therapies rely heavily on identification of modifier genes involved in a particular disease phenotype, which may prove challenging and possibly costly.^46^

Development of modifier gene therapies has expanded rapidly for systemic diseases, including neuromuscular disease, cystic fibrosis, spinal muscular atrophy, and cancer, as well as for retinal disease. Previous studies have shown that modifier genes, such as nuclear hormone receptors, have the potential to “reset” various networks related to retinal disease phenotypes, such as photoreceptor development, phototransduction, metabolism, cone cell development, inflammation, and cell survival, thus restoring the homeostasis in the retina to a healthy state.^47^^,^^50^^–^^53^ One modifier gene therapy, OCU400 (Ocugen, Inc.), is a nuclear hormone receptor-based gene therapy currently in clinical trials to treat retinitis pigmentosa (NCT05203939). This gene agnostic approach has the potential to significantly reduce the need to develop a product for every mutation, thus reducing development and commercialization costs, while expanding the patient reach to address this unmet medical need in rare genetic disease spaces.

## Gene Delivery Approaches in the Retinal Space

**Figure 3.**
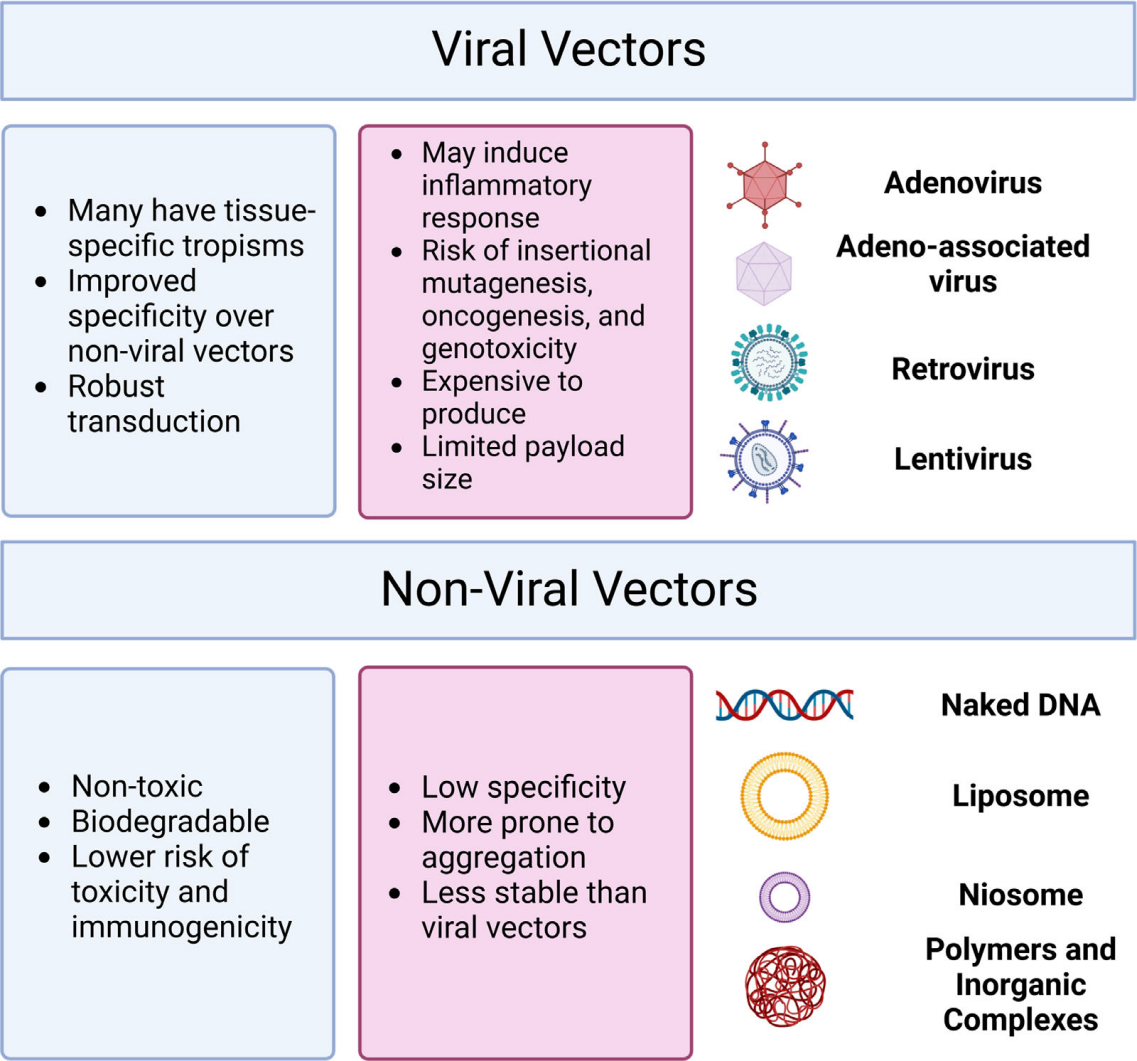
Gene Therapy Vectors may be Either Viral or Non-Viral, Each With its own Advantages and Risks (Created With BioRender.com).

Gene delivery to the retina involves several factors, such as the optimal route of administration, the size of the gene, the immunogenicity and specificity of the vector, and manufacturing complications, including the cost and the ability to scale up production. Various viral and non-viral vectors provide multiple options (Fig. 3, Table 2).^15^

**Table 2.** Gene Therapy Vectors

Vector	Advantages	Limitations	Examples
**Viral vectors**			
Adenovirus (Ad)	Non-integrating Larger payload size (30 kb) Good safety profile Robust transduction in dividing and non-dividing cells Easy to scale. Tissue-specific tropism	Can induce higher inflammatory response than other viral vectors Less durable expression Rapidly cleared due to presence of neutralizing antibodies	Targeting retinoblastoma with oncolytic adenovirus VCN-01^63^ Ad vectors have fallen out of favor due to the robust immune response that causes elimination of the transduced cells^62^
Adeno-associated virus (AAV)	Tissue-specific tropism Weak immunogenicity Non-replicating in nature Transduce proliferating and post-mitotic cells	Transduction limited to bleb area of retina Limited payload size	Luxturna (Spark Therapeutics)
Retrovirus/Lentivirus	Allows direct modification of patient's genome Good candidate for monogenic disease	High risk of insertional mutagenesis/genotoxicity Risk of oncogenesis	RetinoStat (Oxford BioMedica, OXB-201)
**Non-Viral Vectors**			
Naked DNA or RNA	Simple Relatively safe Fast to produce No risk of genotoxicity	Quickly degraded Low specificity Limited uptake	Macugen (pegaptanib; Eyetech/Pfizer) Fovista (pegpleranib; Ophthotech) Zimura (avacincaptad pegol; IVERIC bio-Inc.)
Lipid nanoparticles and liposomes	Non-toxic Biodegradable	Limited stability Prone to aggregation May induce retinal toxicity	QPI-1007 siRNA (Quark Pharmaceuticals) Cai, X. et al. 2010^84^ Han, Z. et al. 2012, 2015^85^^,^^86^ Caracciolo, G. et al. 2008^156^ Mochizuki, S. et al. 2015^157^ Amadio, M. et al. 2016^87^
Niosomes	More stable than liposomes Non-toxic Biodegradable Inexpensive	Efficacy is dependent on route of administration	Al Qtaish et al. 2020^96^ Durak et al. 2020^97^ Mashal et al. 2019^98^ Mashal et al. 2017^99^ Puras et al. 2014^100^ Villate-Beitia et al. 2018^101^
Polymers	Reduced immunogenicity Relatively stable High transfectivity Higher payload size	Potential for cytotoxicity Some polymers may be less stable	Liao et al. 2007^158^ Mastorakos et al. 2015^159^ Kurosaki et al. 2013^160^

### Viral Vector Delivery

Adenoviral vectors derived from the human Ad2 and Ad5 serotypes were some of the first viral delivery systems to be used in retinal gene therapy research.^5^^,^^29^ These viruses show good safety when administered to target tissues, do not integrate in the recipient genome, successfully transduce retinal cells, and can carry genes of around 30 kb long.^4^^,^^29^^,^^54^^–^^56^ However, these vectors tend to be rapidly cleared due to the presence of neutralizing antibodies from pre-existing immunity.^4^^,^^29^^,^^54^^–^^56^ Second generation adenovirus vectors have early gene regions (E2a, E2b, or E4) deleted to reduce viral replication and immunogenicity in the host cells.^29^^,^^57^^–^^61^ Despite these advances, adenovirus vectors are typically not used for retinal gene therapy and targeting retinoblastoma with oncolytic adenovirus VCN-01is the only Ad vectored clinical trial in the ocular field.^62^^,^^63^ The AAV vectors are a common vector choice in ocular and non-ocular gene therapies, offering benefits such as a long duration of transgene expression, extremely low risk of insertional mutagenesis, only a mild inflammatory response, and low possibility of germline transmission.^29^^,^^54^ AAV vectors offer a high number of tissue-specific serotypes, including retina-specific serotypes AAV1, 2 (Luxturna), 4, 5, 6, 7, 8, and 9,^64^ for the treatment of LCA, is based on an AAV2 vector system, and AAV4 and AAV5 are also commonly used for retinal gene therapy due to their specificity to retina and RPE in animal models.^14^^,^^65^ Serotypes may also influence which cell types are transduced, for example, all the retina-specific serotypes are known to transduce RPE, their ability to transduce photoreceptors varies by AAV.^64^ Recombinant AAV (rAAV) vectors have been developed to combine desirable tropisms from multiple serotypes,^66^ or to reduce immune responses or antibody neutralization.^29^ Recent developments use dual/multiple vector strategy^29^^,^^67^ that bypasses the 4.7 kb maximum size restriction for AAVs utilizing inteins and exteins to join multiple peptide products into the large functional protein in the host cells.^68^^–^^72^ There are four notable approvals of gene therapy products using AAV vector systems: Glybera, Luxturna, Zolgensma, and Hemgenix of which only Luxturna (voretigene neparvovec-rzyl), is a retinal gene therapy product to treat LCA. Retroviruses and lentiviruses have been used in several gene therapy products, specifically RetinoStat (Oxford BioMedica, OXB-201) against wet (neovascular)- AMD (NCT01301443), and in stem cell therapy.^3^ However, they carry risks for insertional mutagenesis and germline transmission and may elicit more inflammatory responses than AAVs.^54^^,^^56^^,^^73^

### Non-Viral Delivery

Non-viral gene delivery has been studied less extensively due to questionable transduction efficiency, durability of effect, and ability to reach the therapeutic expression levels. The simplest of the non-viral methods is physical delivery, such as the injection of naked plasmid DNA, siRNA, mRNA, or miRNA,^74^ which shows limited uptake due to quick degradation.^75^ Aptamer-based therapies, such as Macugen (pegaptanib; Eyetech/Pfizer) that received approval for clinical use have since fallen out of favor as other, more effective therapies reached the market.^76^^,^^77^ Zimura (avacincaptad pegol; IVERIC bio Inc.), a complement C5 protein inhibitor is in clinical trials for the treatment of geographic atrophy (NCT04435366 and NCT05536297) and Stargardt disease 1 (NCT03364153).^77^ Other potential methods have included the use of electroporation, gene guns, ultrasounds, and magnetofection, each using physical methods to deliver the target genes to the appropriate locations.^75^ Additionally, modifications of RNA treatments during production, such as use of chemical methods of delivery, can greatly increase their longevity.^78^

Chemical methods of non-viral gene delivery are appealing for their reduced immunogenicity, ease of scaling, reduced expenses for production, and increased payload size.^79^ Inorganic nanoparticles may include metals such as iron or gold, inorganic cations including magnesium or calcium ions, or ceramics such as phosphate or carbonate salts.^75^^,^^80^^,^^81^ Single-molecule DNA nanoparticles compacted by polyethylene glycol (PEG)-substituted lysine peptides (CK30-PEG) have been used to carry payloads up to 20 kb in size,^82^^,^^83^ with several groups demonstrating their safety in BALB/c mice and *rds^+/^^−^ Rho*-/- or *Abca4*-/- mouse models of retinal degeneration.^82^^–^^86^ Similarly, lipid-based transfection systems have shown success in their ability to transfer the target genes into retinal cells in several studies, and multiple lipid-based drugs for eye diseases are already available in the clinic^35^^,^^79^^,^^87^^–^^91^ and for the delivery of CRISPR or ribonucleoproteins for base editing.^92^^,^^93^ Niosomes, which are comprised of uncharged single-chain surfactant and cholesterol^94^^,^^95^ have also shown limited success potential as non-viral gene delivery systems^96^^–^^101^ due to improved transfection efficiency, biocompatibility, and cellular uptake, in addition to effects on intracellular trafficking.^102^ Polymer systems, such as chitosan, hyaluronic acid, polyethylenimine (PEI), poly(amidoamine) (PAMAM), PEG, poly (lactic-co-glycolic acid) (PLGA), and poly(L-lysine) (PLL), have been studied in ocular gene therapy.^35^^,^^75^

## Gene Therapy Challenges

Some of the very traits that make the retina an ideal candidate for gene therapies (i.e. immune privilege) can also introduce additional challenges to gene delivery, such as identification of the disease-causing gene or mutation, ensuring targeted delivery of the product, appropriate route of administration, feasibility in the clinic, and immune responses to the product that may exacerbate already fragile tissue. Simply delivering the product to the desired tissue can be a physical challenge, both due to the isolated nature of the eyes and the delicate nature of diseased tissue. These challenges remain for all products and clinical trials, and several different approaches have been developed to address them.

### Identification of Disease-Causing Genes

One of the biggest problems facing traditional gene therapy and the gene editing approach is their reliance on accurate genetic diagnosis and the prohibitive cost of generating gene-specific therapies. In many inherited retinal diseases, such as RP, hundreds of mutations in many genes may lead to the same clinical phenotypes, limiting the use of a single gene therapy product to only those patients with a confirmed genetic diagnosis in that gene. However, many patients lack a genetic diagnosis altogether; the gene or genes responsible for their disease phenotype have not been identified, removing the possibility of a traditional gene therapy approach.

#### Mutation Agnostic Approaches to Gene Therapy

Modifier gene therapy has the potential to alter the retinal disease state even in the absence of a genetic diagnosis or in the absence of a gene therapy specific to the mutated gene in one of these patients.^47^^,^^50^^–^^53^ These therapies affect various networks related to retinal disease phenotypes rather than directly relying on replacing or modifying the diseased gene and have the potential to “reset” these networks to restore homeostasis of a healthy retina. As with other gene therapies, vector selection and route of administration can influence the efficiency of transduction and effectiveness of the therapy, as well as influencing any immune responses, and so careful design of delivery is just as important using this model while offering a wider potential for treatment.

In addition to modifier therapy and gene editing, therapies that focus on protection of degenerating photoreceptors and RPE cells do not rely on knowledge of a disease-causing genetic mutation. Neuroprotective factors that support the survival of retinal cells, such as glial cell-derived neurotrophic factor (GDNF), ciliary neurotrophic factor (CNTF), brain-derived neurotrophic factor (BDNF), basic fibroblast growth factor, and pigment epithelium-derived factor (PEDF) have shown promise in mouse models of retinal degeneration.^67^^,^^103^^–^^105^ Although repeated administration of these factors directly may lead to inflammation and patient compliance issues, a stable expression of these factors in the retina using a gene therapy approach may offer a similar advantage as modifier therapy in promoting a healthy state in the retina.^67^ Supporting the promise of this method, AAV delivery of BDNF and its receptor^105^ or CNTF^106^ promoted survival and function of retinal ganglion cells in models of optic nerve crush or retinal ganglion cell degeneration in a model for experimental glaucoma, respectively. Similarly, PEDF offers anti-angiogenic and anti-inflammatory effects that may be useful in wet AMD, and a combination treatment of AAV-delivered PEDF with a microRNA to inhibit VEGF successfully reduced choroidal neovascularization in a mouse model of AMD.^107^ MicroRNAs themselves have similar influences as neuroprotective factors on protection of photoreceptors and pathogenesis of retinal disease, and several different microRNAs have been tested in rodent models for their ability to regulate retinal physiology in models for diseases such as AMD.^67^^,^^107^^–^^109^

Optogenetics, a tool that involves the delivery of light-sensitive microbial opsins to retinal cells, may be particularly useful in advanced cases of retinal disease in which photoreceptors have severely degenerated. It has already shown promise in preclinical rodent and nonhuman primate (NHP) models, as well as advancing to the clinical trial stage for several products, and provides new photosensitive genes, such as channel rhodopsin, halorhodopsin, and melanopsin, to existing neural networks.^67^ However, optogenetics still require optimization to allow for complex visual processing and to increase the sensitivity of the proteins that are currently used.^10^^,^^15^ Despite this challenge, optogenetic gene therapies are currently in clinical trials (Table 4).

### Gain-of-Function Mutations

Another difficult challenge is addressing toxic gain-of-function mutations, as observed in autosomal dominant RP and mutations to *RHO*. Similarly, a dominant negative mutation can lead to toxic loss of function. In cases of these toxic gain or loss of function, additional gene silencing methods can been attempted, including the use of short hairpin RNAs and allele-specific ribozymes, but this would lead to complete silencing of the gene; the challenge remains to provide a version of the gene that is not susceptible to the silencing treatment.^15^ Gene editing techniques, such as the CRISPR/Cas9 systems, could also provide solutions to these mutations.

### Effective Targeting

One of the greatest challenges to gene therapy is ensuring the product reaches the tissue or cells of interest. For example, the blood-retina barrier (BRB) can prevent a gene therapy given systemically from reaching the retina. Additionally, the gene therapy product must be able to transduce the diseased cells themselves. The use of specific vectors or cell-specific promoters can increase the efficiency of targeted interactions, and directly administering the product to the target tissue can increase its effectiveness.

#### Vector Selection and Targeted Engineering

Adenoviruses and AAVs are known to have tissue-specific tropisms that make certain serotypes attractive for development of retinal gene therapy products.^14^^,^^29^^,^^56^ Due to their tissue-specific tropisms, some of the most common AAV serotypes in use for retinal gene therapy include AAV2, AAV4, AAV5, and AAV8, as mentioned previously.^26^^,^^110^ The use of recombinant or engineered vector capsids can further increase tissue-specific targeting by allowing more precise control over the various elements in a capsid, and vector modifications. Selection of cell-type-specific promoters, further refines targeting to the affected cell types.^29^^,^^111^

RPE cells can be targeted using AAV1, AAV4, or AAV6 vectors or with the engineered AAV2-7m8 vector, and photoreceptors and Müller glial cells are targeted by serotypes AAV2, AAV5, AAV6, AAV7, AAV8, and AAV9 as well as engineered AAV2-7m8 and AAV8BP2 vectors.^56^^,^^112^^–^^116^ One group recently used a promoter engineering approach to create an extensive library of 230 AAVs that each contained a different synthetic promoter designed specifically to target cell types.^111^

#### Route of Delivery

**Figure 4.**
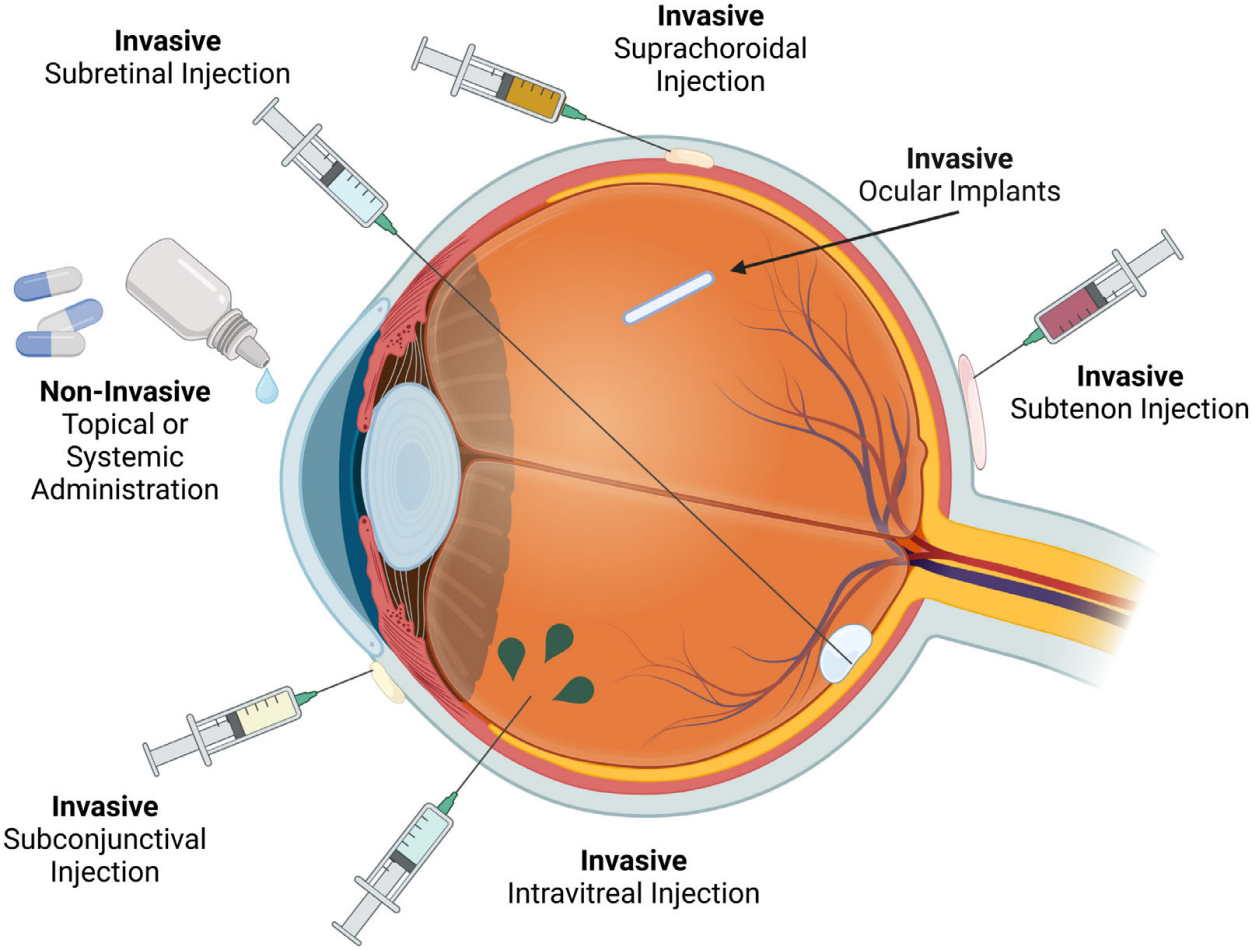
Ocular routes of administration may be invasive or non-invasive (created with BioRender.com).

Once the appropriate vector design has been determined, researchers still have the challenge of finding the optimal route to deliver the gene therapy to the tissue. Gene therapies can be delivered to the retina by ocular or systemic routes (Fig. 4, Table 3), although generally it is preferred to use a route that will bring the therapy as close to the target tissue as possible, such as subretinal injections for outer retina targets and intravitreal injections for inner retina targets.^56^ Systemic administration has the advantage of convenience, but the lack of specific targeting can lead to nonspecific effects in non-ocular tissues, reduced bioavailability in the target tissue, and an increase in the risk of immunogenicity as more of the body is exposed to the therapy.^35^ Ocular administration restricts a gene therapy's effect to the target tissue and reduces immunogenicity. Invasive ocular delivery methods offer more targeted delivery and therefore increase the bioavailability of drug products, but these methods increase the risk of complications, such as infection, retinal detachment, and hemorrhage. These procedures typically require skilled and experienced surgeons for successful administration.^117^ Noninvasive delivery methods offer drug delivery with fewer procedure-related complications and easier administration but with reduced bioavailability.^35^^,^^118^ Therefore, determining the optimal route of delivery for a specific gene therapy product is critical for efficient transduction and minimized immune response in patients.

**Table 3.** Ocular Routes of Administration

Type		Advantages	Limitations	Examples
Noninvasive	Topical (e.g. eye drops, contact lenses)	No physical disturbance to eye Easy administration	Reduced bioavailability Increased clearance	Latanoprost for glaucoma
	Systemic	No physical disturbance to eye Easy administration	Reduced bioavailability Off-target effects	Vitamin regimens for ocular diseases
Invasive	Subtenon	Can avoid complications from needle injections by using a cannula	Causes physical damage to eye structures Increase in ocular pressure	Steroid injection to treat chronic uveitis in the posterior segment
	Subconjunctival	Improved bioavailability over topical methods Good for anterior and posterior portions of the eye	Variable absorption Systemic absorption may occur	Antibiotic or corticosteroids to treat lesions in the anterior segment
	Ocular implants	Extended release of product Can be biodegradable	Nonbiodegradable implants must be surgically removed	Ozurdex (dexamethasone) to treat retinal vein occlusion
	Subretinal	Direct delivery to retinal cells	Requires trained surgeons	Luxturna
	Intravitreal	Less invasive than subretinal Easy surgery Widespread distribution	May have reduced transduction of retina and RPE Potential for particles or floaters	Anti-VEGF treatments for AMD
	Suprachoroidal	Does not require retrobulbar anesthesia in an operating room Better bioavailability than intravitreal	Requires trained surgeons Procedure-related complications Faster clearance	Xipere for macular edema secondary to posterior uveitis

##### Noninvasive Delivery

Noninvasive delivery methods include topical administration, such as eye drops, iontophoresis, ultrasonics, transdermal systems, and contact lenses. These methods avoid the risk of surgically induced damage to parts of the eye but may also reduce the bioavailability of the therapeutic. Of the noninvasive methods, topical administration is the most used.^35^^,^^118^ This method avoids complications of first-pass metabolism and has the advantage of easy administration, sometimes by the patients themselves. However, barriers, such as the pre-corneal tear film, the structure of the cornea, limited volume, lacrimal drainage system, reflex tearing, and aqueous outflow of the eye, reduce the bioavailability of the product to around 5% or less.^35^^,^^118^^,^^119^ Iontophoresis uses a low electrical current to allow easier penetration of ionic drugs into tissues. Placement of the electrode can target different regions of the eye but care should be taken as higher currents can cause tissue damage.^35^^,^^119^^–^^121^ Ultrasonic devices use a sound field above 20 kHz to improve penetration of a product through skin and eyes, whereas transdermal patches use controlled longer release that may be beneficial for chronic ocular disease.^35^^,^^119^ Finally, contact lenses may be soaked in a drug solution before application to the eyes, thus serving as an alternative to eye drops and improving the sustained release of transdermal patches. Contact lenses tend to have better compliance and fewer systemic side effects than other methods, but they are limited by the solubility of the product, the time needed to soak the lens, and the amount of drug that is discarded following soaking.^35^^,^^122^

##### Uncommon Invasive Routes of Delivery for Gene Therapy

Some invasive delivery methods less commonly used in gene therapy include ocular implants, subtenon, and subconjunctival injections.^35^^,^^118^ Subtenon injections have been used to treat diabetic macular edema and surgery-related choroidal detachments, but they are occasionally associated with increases in intraocular pressure.^123^^–^^125^ Subconjunctival injections are dependent on the size of the particles. Although they demonstrate improved bioavailability over topical administration, systemic absorption may occur.^35^ Biodegradable and non-biodegradable ocular implants have been used in several drug treatments for conditions such as diabetic macular edema and posterior uveitis, among others, and provide a method for sustained release of poorly soluble steroids.^118^^,^^126^

##### Subretinal Injection

Subretinal drug is administered between the retinal photoreceptor cells and the RPE layer, requires less vector than intravitreal injections to achieve a therapeutic effect on the retina.^127^ However, this route of administration can further damage an already damaged retina, leading to retinal or RPE detachment from underlying layers, hemorrhage, and changes in retinal pigmentation.^35^^,^^127^^,^^128^ Injections affecting the thinner and more fragile fovea carry greater risk than other retinal regions.^15^ However, subretinal injections have not been associated with severe inflammation following treatment with AAV8, AAV2, or AAV5, increasing its appeal as a route of administration.^19^^,^^26^ The use of corticosteroids is typical to reduce procedure-related inflammation and immune responses to the vector.^27^ Targeted engineering of vector capsids may also alleviate inflammation and the use of robotic devices may alleviate the drift and error of human surgeons in the operating room.^19^

##### Intravitreal Injection

Intravitreal injections are the least invasive of these surgical administration methods for gene therapy products targeting the inner retina. Anti-VEGF therapies for AMD and diabetic retinopathy typically use intravitreal injections, for the repeated injections. Despite these advantages, intravitreal injections also carry risks of adverse reactions, including inflammation, endophthalmitis, increased intraocular pressure, retinal detachment, hemorrhage, and cataracts, and due to the repeated use of these injections in various treatments, patient compliance can become an issue.^119^^,^^127^^,^^129^ Most gene therapy viral vectors given intravitreally, fail to adequately transduce photoreceptors and RPE, due to the inner limiting membrane that divides the vitreous from the retina.^1^^,^^15^^,^^119^^,^^127^ However, development of recombinant vectors AAV2.GL and AAV2.NN improve the retinal delivery of a gene therapy product after intravitreal injection.^56^^,^^66^ Increased non-ocular biodistribution of ranibizumab or bevacizumab have been observed with the intravitreal route causing systemic adverse events like ecchymosis, gastrointestinal and vaginal hemorrhage, hematoma, increased blood pressure, cerebrovascular accident, myocardial infarctions, iliac artery aneurysm, and death.^26^^,^^129^ Intravitreal injection is also known to be more immunogenic than subretinal injection.^27^ Adverum Biotechnologies terminated the development of ADVM-022, an intravitreal AAV-based aflibercept gene therapy for AMD and DME due to dose-limiting toxicity in multiple patients.^130^

##### Suprachoroidal Injection

Suprachoroidal injections, administered between the choroid and sclera, do not require vitreoretinal surgeries, and show better bioavailability than intravitreal injections.^1^^,^^131^^,^^132^ This injection requires a skilled surgeon and must be performed in an operating room. Procedure-related adverse events include suprachoroidal hemorrhage, endophthalmitis, choroidal tears, changes in choroidal blood flow, inflammation, and retinal detachment.^131^

### Immunogenicity and Retinal Toxicity

Despite many advancements in the overall safety profiles and quality assurance of gene therapy products, there remain concerns regarding the safety of gene therapy products, particularly at high doses. Although there have been safety issues observed in gene therapy trials outside the eyes, few serious adverse events directly related to recombinant AAV gene therapies have been reported in ocular gene therapy trials.^26^ The combination of the procedure and the retinal gene therapy drug carries the potential for toxicity to the retina, which is already fragile due to the disease.^128^^,^^133^ Adverse events include inflammatory responses, increases in intraocular pressure, loss of retinal layers, decrease in electroretinography (ERG) amplitudes, antidrug antibody (ADA) responses to vector, and toxicity in the photoreceptors and RPE layers.^23^^,^^26^^,^^134^^–^^138^ Elevated immune responses due to defects in blood-tissue barriers lead to production of neutralizing antibodies that limit the effectiveness of gene therapy.^133^^,^^139^ Adenoviral vectors tend to provoke rapid neutralizing antibody and immunogenic responses, but these responses are reduced with improved targeting of the tissues of interest.^4^ Vector sequences like CpG sequences, and CMV and CAG promoters can also lead to inflammatory immune reactions, which can trigger the activation of microglia in the retina and may exacerbate degeneration.^26^^,^^140^ Immunogenicity against the viral vector that limits gene therapy efficacy is a major concern.^4^^,^^21^^,^^25^^,^^26^^,^^141^ and is one of the biggest exclusion criteria in clinical trials.^14^^,^^26^ In addition to neutralizing antibodies, cell based immune responses have often been observed at higher doses (above 1 × 10^11^ vg) in many ocular gene therapy trials.^25^^,^^26^ Future products will need to optimize the balance between the effective dose level and the potential for toxic effects. However, Luxturna's success demonstrates that successful gene therapy for retinal disease is possible.

In the clinical setting, screening patients for presence of neutralizing antibodies for the gene therapy vector can improve outcomes.^14^^,^^26^^,^^142^ Alterations to the product itself, by changing immunogenic sequences, would alter how a product will interact with immune cells in vivo. Assessments of immunogenicity both before and after use in a clinical setting, must be continuously monitored and^143^ modulating CD4^+^ T cell and B cell responses may assist in reducing ADAs.^144^ For example, immature dendritic cells (iDCs) are antigen presenting cells (APCs) that often present self-antigens without stimulating T cell responses, which may allow for tolerance of peptide sequences that could be harnessed in biotherapeutic treatments.^143^^–^^145^ Predicting and removing epitope sequences that can stimulate T or B cells offers another opportunity to reduce immunogenicity.^144^^,^^145^ Epitopes can also be shielded by polymers, such as PEG, XTEN, or PAS, or by methylating, glycosylating, or bio conjugating biotherapeutics, to effectively hide the surface epitopes of the product from the immune system or inducing tolerance to the antigen.^145^^,^^146^

Besides altering the product directly, co-treatment with anti-inflammatory or anti-NAb substances may reduce the immune response to a gene therapy. Minocycline treatment has been shown to reduce inflammatory cytokine expression in the retina.^147^ Administration of the IgG-cleaving endopeptidase imlifidase (IdeS) or co-administration of a synthetic vaccine particle containing rapamycin with AAV treatment have both been shown to reduce immune cell activation and decrease titers of anti-AAV antibodies.^148^^,^^149^ Corticosteroid treatments are also often used to minimize the effects of inflammation and cytokine expression that occur because of administration procedures, reducing the immune response in the ocular space.^150^^,^^151^ Future gene therapy vectors and clinical studies may rely on one of these methods or a combination of approaches to improve the safety profile and efficacy of a product by reducing or eliminating immunogenicity, making safe and effective treatments with few adverse effects possible.

### Animal Model Selection

Rodent models are often appealing for proof-of-concept and efficacy studies, as many of the diseases under study have genetic rodent models available. However, there are other cases in which genetic models are not available or may not fully reflect a disease, such as the physical damage models sometimes used for AMD studies. These models may include laser ablation of Bruch's membrane, as in the choroidal neovascularization model in rats, or oxygen-induced retinopathy models. Although these models mimic some hallmarks of the disease, they do not fully capture human pathophysiology and viral vector serotype affinity for certain tissues may not translate well from small mammal models to NHPs or humans.^14^ The closest model to a human is NHP, which has eye anatomy and physiology like humans and may provide the closest response to what could be expected in the clinic.^152^ However, the use of NHP models may have serious concerns, including ethical issues, limited availability of animals, and the high cost of performing NHP studies. Several studies in the Luxturna trials failed to show the same effects in humans as had been observed in animal models, such as a lack of improvement in full field electroretinograms in humans that had been seen in dogs and a decline in vision improvement that had not been observed in the animal models.^15^ Researchers must consider how closely the model resembles the human counterpart in anatomy (such as size, volume, and critical structures including the macula), physiology, and sequence homology between the target human protein of interest and the model's homolog, which can impact factors such as immunogenicity.

### Manufacture of Gene Therapy Products

Beyond efficacy and safety concerns, gene therapy products have several challenges in manufacturing and production cost of the final clinical products. Although AAVs are most widely used vectors in retinal gene therapy, they remain difficult and costly to produce.^16^^–^^18^ Production methods are difficult to scale from the bench to a commercial size, often with lower yields and efficiency.^17^ Manufacturing methods face complications, such as ensuring product potency, purity, and quality. There are considerable challenges in characterization testing, such as separating empty and full capsids.^153^ Aside from manufacturing challenges, AAV gene therapy products also face challenges in clinical applications. Although quite stable, AAVs often must be stored at -80°C for long term. Viral vectors may need to be diluted to the required dosage at the clinical site, requiring the facility to have the appropriate biological safety cabinets systems in place. Stability of gene therapy products must be closely monitored, as once the product is thawed, there is limited time for clinicians to use it.

## Ongoing Gene Therapy Clinical Development and Approved Gene Therapy for Retinal Diseases

Luxturna (voretigene neparvovec-rzyl; Spark Therapeutics, Inc.) was the first gene therapy approved in the United States to treat biallelic *RPE65* mutation-associated retinal dystrophy. Since this first approval, 24 additional cell and gene therapies have been approved by the FDA for other non-retinal diseases (fda.gov). Presently, there are more than 40 active clinical trials or follow ups for ocular diseases using AAV vectors alone.^154^ In the United States, over 1000 gene therapy studies are active for a wide range of diseases and conditions. Of these, more than 30 have indications for retinal diseases and are either actively recruiting or enrolling by invitation (Table 4). Many of the gene therapy trials in progress use gene replacement platforms with AAV vectors. However, more studies are considering the use of CRISPR/Cas9 systems, such as Editas Medicine, which is targeting LCA10 caused by *CEP290* mutations, or modifier therapy, such as Ocugen, Inc., which has a product (OCU400) in clinical trials for retinitis pigmentosa associated with mutations in *NR2E3*, *RHO*, and *CEP290* genes. The OCU400 modifier gene product has the potential to address multiple genetic mutations associated with RP and LCA and could be a potential mutation agnostic therapy if it demonstrates success in human clinical trials.

**Table 4.** Summary of Active Gene Therapy Clinical Studies with Ocular Disease Indications

Condition	Product	Vector	Route of Administration	Clinical Phase	Sponsor	NCT Numbers
Achromatopsia	AGTC-401	rAAV2	Subretinal	1/2	Applied Genetic Technologies Corp.	NCT02599922
	AGTC-402	rAAV2	Subretinal	1/2	Applied Genetic Technologies Corp.	NCT02935517
Dry age-related macular degeneration	GT005	rAAV	Subretinal	1/2	Gyroscope Therapeutics Limited	NCT03846193
Wet age-related macular degeneration	ADVM-022	AAV.7m8	Intravitreal	2	Adverum Biotechnologies, Inc.	NCT05536973
	RGX-314	AAV8	Subretinal	2/3	REGENEXBIO Inc.	NCT04704921 NCT04832724 NCT05407636
	4D-150	AAV	Intravitreal	1/2	4D Molecular Therapeutics	NCT05197270
Geographic atrophy	Zimura (avacincaptad pegol)	NA (aptamer)	Intravitreal	3	IVERIC bio, Inc.	NCT04435366 NCT05536297
Choroideremia	BIIB111	AAV2	Subretinal	3	NightstaRx Ltd, a Biogen Company	NCT03584165
	4D-110	Capsid Variant 4D-R100	Intravitreal	1	4D Molecular Therapeutics	NCT04483440
Diabetic macular edema	ADVM-022	AAV.7m8	Intravitreal	2	Adverum Biotechnologies, Inc.	NCT04418427
Diabetic retinopathy	RGX-314	AAV8	Suprachoroidal	2	REGENEXBIO Inc.	NCT04567550 NCT05296447
Autosomal recessive Leber's congenital amaurosis	SAR439483	AAV5	Subretinal	1/2	Atsena Therapeutics Inc.	NCT03920007
Leber's congenital amaurosis	EDIT-101	Gene editing via CRISPR/ Cas9	Subretinal	1/2	Editas Medicine, Inc.	NCT03872479
	AAV2/5-OPTIRPE65	AAV2/5	Subretinal	Follow-up	MeiraGTx UK II Ltd	NCT02946879
	AAV2-hRPE65v2	AAV2	Subretinal	1/2; 3; follow-up	Spark Therapeutics	NCT01208389 NCT03597399 NCT00999609 NCT03602820
Leber hereditary optic neuropathy	scAAV2-P1ND4v2	AAV2	Intravitreal	1	Byron Lam	NCT02161380
	GS010	rAAV2/2	Intravitreal	3	GenSight Biologics	NCT03293524
Non-syndromic retinitis pigmentosa	GS030-DP with medical device GS030-MD	AAV2	Intravitreal	1/2	GenSight Biologics	NCT03326336
Retinitis pigmentosa	BS01	rAAV	Not specified	1/2	Bionic Sight LLC	NCT04278131
	AAV2/5-hPDE6B	AAV2/5	Subretinal	1/2	Coave Therapeutics	NCT03328130
	vMCO-010	AAV2/5	Intravitreal	2	Nanoscope Therapeutics Inc.	NCT04945772
	OCU400	AAV5	Subretinal	1/2	Ocugen	NCT05203939
	QR 421a	RNA antisense oligonucleotide	Intravitreal	2	ProQR Therapeutics	NCT05085964
	rAAV.hPDE6A	rAAV	Subretinal	1/2	STZ eyetrial	NCT04611503
X-linked retinitis pigmentosa	AGTC-501	rAAV2	Subretinal	1/2	Applied Genetic Technologies Corp	NCT03316560
	AAV5-RPGR	AAV5	Subretinal	3	MeiraGTx UK II Ltd	NCT04671433
	AAV5-RPGR	AAV5	Subretinal	3	MeiraGTx UK II Ltd	NCT04794101
	BIIB112	AAV8	Subretinal	3	NightstaRx Ltd, a Biogen Company	NCT03584165
	4D-125	Capsid Variant 4D-R100	Intravitreal	1/2	4D Molecular Therapeutics	NCT04517149
X-linked retinoschisis	rAAV-hRS1	rAAV2	Intravitreal	1/2	Applied Genetic Technologies Corp	NCT02416622
	AAV-RS1	AAV8	Intravitreal	1/2	National Eye Institute (NEI)	NCT02317887
Stargardt disease	vMCO-010	AAV2	Intravitreal	2	Nanoscope Therapeutics Inc.	NCT05417126
	Zimura (avacincaptad pegol)	NA (aptamer)	Intravitreal	2	IVERIC bio, Inc.	NCT03364153

## The Future of Gene Therapy

Over the last 25 years, great strides have been made in retinal gene therapy, and each new development leads toward improved products with better safety and efficacy. However, there is still a significant need for these products to reduce the high healthcare costs, economic burden, and costs to affected individuals and families and to improve patient well-being and quality of life.^155^ As the field continues to grow, research and drug developers are likely to focus on ways to improve the vector design to improve efficiency, open additional vector targets, and increase overall product safety. Additionally, standardized methods for manufacturing may reduce variability of products between batches which would also be likely to improve safety and efficacy.

An important consideration for future gene therapy product development is finding and implementing methods to treat genetic disease in the absence of a genetic diagnosis or to treat multiple mutations with a single product. These methods would address two persistent problems in gene therapy: the high cost of production and the need to identify the genetic basis of a disease from individual to individual. A product to address both issues would allow for greater distribution among patient populations and improved cost to drug developers. Identification of mutation agnostic modifier genes and protective factors that may modulate the various phenotypes of genetic retinal diseases and continued development of optogenetic technology will open new avenues for treatment across a wide range of conditions.

Improvements to vector design are also likely to focus on aspects such as reducing immunogenicity, improving target specificity, and improving transduction efficiency. Although AAVs have been the vector of choice in recent years, less immunogenic options, such as engineered viral vector capsids, lipid-based nanoparticles or polymer systems, may prove important to future vector design. Recombinant vectors already use some of these modifications, and future products are likely to continue this trend. Future gene therapies may also find ways around the payload size limitations of vectors, such as use of non-viral techniques or dual or triple transduction strategies.

As gene therapy grows in use, availability of appropriate materials for viral vector purification and harvest will need to increase. In addition, standardized methods will continue to develop and become accepted across manufacturers to improve product quality and cost-effectiveness. Some of the current limitations for product quality and efficiency are being addressed as new technologies emerge, such as a shift from traditional quantitative PCR (qPCR) methods to droplet digital PCR (ddPCR) methods during in-process and release testing, changes in vector production strategies from a standard triple plasmid transfection model to a plasmid-free production system, such as tetracycline-enabled self-silencing adenovirus (TESSA), that may reduce the possibility of in-process contaminants, and changes from batch production to a perfusion-based continuous production process to improve yield. Changes in the vectors of choice may also require shifts in acceptable manufacturing practices and adaptation of existing technology.

With the advances that have so far been made in retinal gene therapy and the progress likely to be made in the coming years, retinal therapy remains a powerful tool for the greater gene therapy field, providing innovative solutions to complex problems and modeling gene therapy in a closed system. Success in treating retinal diseases, such as the approval of Luxturna, offers hope for technology, bringing a possibility of treatment, improved quality of life, and even a cure to thousands of patients worldwide. Regardless of the research yet to be done, it is a focus on the well-being and outcomes of patients that will continue to drive the many improvements yet to be made, and both researchers and drug developers should keep this in mind as they move the field forward.
